# Large Tubulovillous Rectal Mass Causing McKittrick-Wheelock Syndrome

**DOI:** 10.14309/crj.0000000000001832

**Published:** 2025-09-10

**Authors:** Danzhu Zhao, Bianca Thakkar, Teresa Da Cunha, Minh Thu T. Nguyen, Alexander Potashinksy

**Affiliations:** 1 Department of Medicine, University of Connecticut, Farmington, CT; 2 Department of Medicine, Division of Gastroenterology and Hepatology, University of Connecticut, Farmington, CT; 3 Department of Medicine, Division of Gastroenterology and Hepatology, The Hospital of Central Connecticut, New Britain, CT

**Keywords:** McKittrick-Wheelock Syndrome, tubulovillous adenoma, secretory diarrhea, electrolyte imbalance, transanal surgical excision

## CASE REPORT

**Figure 1.**
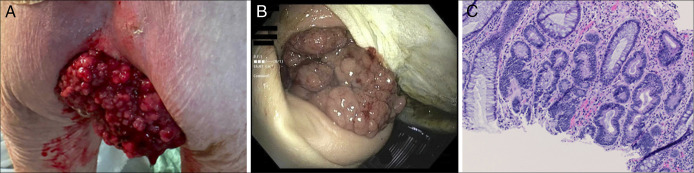
(A) Digital rectal examination revealed a nodular rectal mass measuring over 10 cm in diameter. (B) View of the nodular rectal mass before colonoscopy. (C) Hematoxylin and eosin stain at 200× magnification showing a tubulovillous adenoma with no evidence of invasion.

We present 2 unique clinical images and an accompanying histologic image from a 74-year-old woman with a severe case of McKittrick-Wheelock syndrome. She had no known medical history and presented with weakness, nausea, decreased oral intake, and clear rectal discharge. Laboratory results revealed hyponatremia (121 mmol/L), hypochloremia (80 mmol/L), hypokalemia (2.2 mmol/L), elevated blood urea nitrogen (47 mg/dL), normal creatinine (0.9 mg/dL), and elevated carcinoembryonic antigen (12.6 ng/mL). Digital rectal examination revealed reducible prolapse and a more than 10 cm nodular rectal mass, 3 cm from the anal verge (Figure 1A). Computed tomography of the abdomen and pelvis showed a distended rectum (9.2 cm × 8.8 cm) with mixed fluid and soft tissue concerning for a neoplasm. Colonoscopy showed multiple sessile polyps (in the cecum, ascending, and transverse colon) and a large and circumferential fungating mass in the rectum (Figure 1B). Pathology revealed tubular adenomas in the colon and a tubulovillous adenoma in the rectum (Figure 1C).

Pelvic MRI showed internal sphincter involvement, indeterminate T stage, and N0. She was treated with fluids and underwent 2 transanal excisions. This case and set of images highlight the remarkable manifestations of McKittrick-Wheelock syndrome, a rare but potentially life-threatening syndrome associated with a large tubular and tubulovillous adenoma.^1,2^

## DISCLOSURES

Author contributions: Drafting manuscript: D. Zhao, B. Thakkar, TD Cunha, MT Nguyen, A. Potashinksy. Manuscript revision, intellectual revisions, mentorship: MT Nguyen, A. Potashinksy. Final approval: D. Zhao, B. Thakkar, TD Cunha, MT Nguyen, A. Potashinksy. D. Zhao is the article guarantor.

Financial disclosure: None to report.

Previous presentation: ACG 2024 Annual Scientific Meeting and Postgraduate Course; October 25–30, 2024; Philadelphia, PA. Abstract P0374.

Informed consent was obtained for this case report.
